# Integrating Omics Data and AI for Cancer Diagnosis and Prognosis

**DOI:** 10.3390/cancers16132448

**Published:** 2024-07-03

**Authors:** Yousaku Ozaki, Phil Broughton, Hamed Abdollahi, Homayoun Valafar, Anna V. Blenda

**Affiliations:** 1 Department of Biomedical Sciences, University of South Carolina School of Medicine Greenville, Greenville, SC 29605, USA; yozaki@email.sc.edu (Y.O.); pxb@email.sc.edu (P.B.); 2 Department of Computer Science and Engineering, Molinaroli College of Engineering and Computing, Columbia, SC 29208, USA; ha25@mailbox.sc.edu; 3 Prisma Health Cancer Institute, Prisma Health, Greenville, SC 29605, USA

**Keywords:** omics technologies, artificial intelligence, cancer, machine learning, deep learning

## Abstract

**Simple Summary:**

Cancer remains one of the leading causes of death worldwide, which emphasizes the need for its early and accurate diagnosis and prognosis. Our review explores AI’s potential in this field, analyzing 89 recent studies from 2020 through 2023. Specifically, these studies included AI applications for the analysis of multi-omics data: radiomics, pathomics, clinical records, and lab data. Notably, eight studies combined diverse omics data types (genomics, transcriptomics, epigenomics, and proteomics). Integration of AI for the analysis of clinical and omics data contributes to a significant advancement and is essential for safe clinical implementation.

**Abstract:**

Cancer is one of the leading causes of death, making timely diagnosis and prognosis very important. Utilization of AI (artificial intelligence) enables providers to organize and process patient data in a way that can lead to better overall outcomes. This review paper aims to look at the varying uses of AI for diagnosis and prognosis and clinical utility. PubMed and EBSCO databases were utilized for finding publications from 1 January 2020 to 22 December 2023. Articles were collected using key search terms such as “artificial intelligence” and “machine learning.” Included in the collection were studies of the application of AI in determining cancer diagnosis and prognosis using multi-omics data, radiomics, pathomics, and clinical and laboratory data. The resulting 89 studies were categorized into eight sections based on the type of data utilized and then further subdivided into two subsections focusing on cancer diagnosis and prognosis, respectively. Eight studies integrated more than one form of omics, namely genomics, transcriptomics, epigenomics, and proteomics. Incorporating AI into cancer diagnosis and prognosis alongside omics and clinical data represents a significant advancement. Given the considerable potential of AI in this domain, ongoing prospective studies are essential to enhance algorithm interpretability and to ensure safe clinical integration.

## 1. Introduction

In 1950, Alan Turing introduced the concept of a thinking machine, marking the birth of artificial intelligence (AI) [1]. Today, AI has seamlessly integrated into our lives through familiar names like Siri, Alexa, and Google Assistant. The impact of AI is profoundly felt in oncology, where it has revolutionized the approach to complex challenges posed by cancer. AI-driven techniques have notably elevated the precision and efficiency of oncologic research, opening doors to personalized cancer treatments. Its applications span various areas, including cancer image analysis, genomic studies, data mining from medical records, and drug discovery [2,3,4,5].

There are two main subsets of AI: machine learning and deep learning [4]. Machine learning is a branch of AI that concentrates on creating computer software or algorithms capable of learning from data to make predictions autonomously, without the need for explicit programming. Three fundamental branches of machine learning are supervised, unsupervised, and reinforcement learning [6]. Supervised learning trains models on labeled data, enabling the algorithm to learn patterns, like differentiating benign and malignant tumors in medical imaging for cancer detection. Unsupervised learning works on unlabeled data, identifying patterns within, like grouping patients based on genetic similarities for personalized treatment plans in cancer research. Reinforcement learning trains models to make sequential decisions, learning through trial and error, optimizing treatment plans in medicine. Meanwhile, deep learning uses neural networks with multiple layers to learn representations of data and excels in handling unstructured data like images and text [7]. Convolutional neural networks (CNN) are phenomenal at image recognition tasks such as cancer image analysis. Recurrent neural networks and long short-term memory networks are frequently utilized in sequential data, aiding in genetic sequence analysis and mining medical records.

Considering the technological advances in the collection of multi-omics data over the past decades, their integration into cancer research is paramount to help us better understand this complex disease. Multi-omics includes several “-omics” methodologies, like genomics, transcriptomics, proteomics, epigenomics, and metabolomics, to comprehensively understand biological systems [8]. Each “-omics” field contributes to deeper insights into biological systems and diseases, unraveling various levels of anatomy and molecular and cellular interactions, laying the groundwork for precision medicine and personalized healthcare approaches. Genomics focuses on an organism’s complete set of genes, gene sequences, interactions, and functions to understand how variations in genes contribute to traits or diseases. Epigenomics examines chemical modifications and alterations in DNA that regulate gene expression without changing the DNA sequence itself to understand their impact on gene activity and cellular functions. Transcriptomics examines all RNA transcripts produced by cells or organisms at a given moment to discover levels and variations in gene expression. Proteomics studies all proteins within cells, tissues, or organisms to understand their biological processes. Metabolomics analyzes the roles of small molecules or metabolites within a biological system. Microbiomics analyzes the collective genetic material of microorganisms in specific environments. Radiomics involves extracting and analyzing quantitative data from medical imaging, like CT scans or MRIs, to identify patterns and correlations between imaging features and diseases such as textures [9]. Pathomics analyzes tissue samples at a microscopic level, integrating imaging, pathology, and molecular data to unravel disease mechanisms and assist in diagnosis and treatments [10]. In this review, we included radiomics and pathomics because they augment the comprehensive understanding provided by multi-omics approaches by supplying vital spatial and structural insights at both the tissue and imaging levels.

In the current era of personalized medicine and precision oncology, providers need to tailor treatment for each patient based on diagnoses and prognoses that are derived from enormous amounts of data. AI enables providers to organize and process the data to achieve goals that cannot be achieved with the human mind alone. Alongside multi-omics, radiomics, pathomics data, and clinical information—encompassing laboratory results and demographic information—play pivotal roles in predictive modeling and personalized treatment. They offer insights into a patient’s physiological status, potential risk factors, and responses to specific interventions, with the aim of tailored cancer management strategies. The fields of multi-omics, radiomics, pathomics, and clinical data analysis with AI have exploded in the past decade but these advances have not been comprehensively reviewed. This review paper aims to close the gap by defining the novel scope in the following way.

## 2. Materials and Methods

We included randomized controlled trials and cohort studies of application of artificial intelligence in determining cancer diagnosis and prognosis using multi-omics data, radiomics, pathomics, and clinical and laboratory data. In this study, the emphasis lies on exploring the potential of AI in cancer prediction and diagnosis rather than on establishing a direct comparison group or intervention. However, in some studies, a comparison group might involve traditional methods of cancer prediction and diagnosis without AI. ChatGPT was utilized in this study to check spelling and grammar errors.

**Figure 1:**
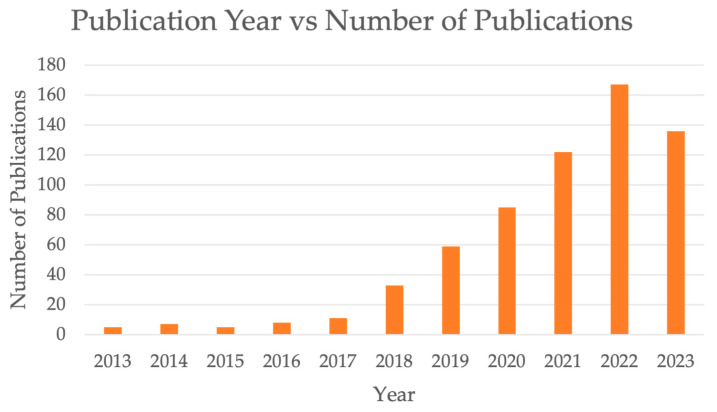
Publication year vs. number of publications.

As a first step, we compiled articles from the past decade to get a better sense of the volume of publications. Among 638 articles as shown in Figure 1, the number of publications on AI and omics from 2018 to 2023 has notably increased, particularly since 2020. However, this query was before we performed screening process and thus included articles from predatory journals. To focus on more recent developments and ensuring relevance to current trends of AI, we opted to narrow the time frame of the studies from 2013–2023 to 2020–2023. It is important to note that the number of publications in 2023 is less than 2022 due to lag in the report of all publications in 2023 by various publishers (delay in complete reports). PubMed and EBSCO databases were used to search the eligible publications from 1 January 2020 to 22 December 2023. The query terms were “artificial intelligence”, “machine learning”, “deep learning”, “cancer diagnosis”, “cancer prognosis”, “multi-omics”, “genomics”, “epigenomics”, “transcriptomics”, “proteomics”, “metabolomics”, “microbiomics”, “radiomics”, “pathomics”, and “clinical data.” Articles on relevant clinical studies in English were included. The search criteria on PubMed were filtered to include only results with “full text available.” On EBSCO, we utilized the “find all my search term” option and included the “also search within full text of the articles” expander. We set result limits to include articles that were peer-reviewed, with full text and references available.

From 638 articles, we selected 212 articles to screen based on the following processes. We used articles from publications with the DOAJ (Directory of Open Access Journals) seal to prevent articles from predatory journals. The DOAJ is a reputable database that indexes high-quality, open-access scholarly journals. We postulated that using articles from journals with the DOAJ seal would add a layer of quality assurance since the DOAJ employs a stringent review process for journal inclusion. We independently screened the database on December 23, 2023, and reached a consensus under the instructions of a project supervisor (Dr. Anna Blenda). A meta-analysis could not be conducted due to the heterogeneity in the design of these studies. We utilized Zotero 6.0.37, reference management software, for the systematic screening of articles throughout the review process. In Zotero, articles were screened by title and abstract. Full texts were retrieved. We reviewed the full texts against the inclusion criteria, which is peer-reviewed, scholarly articles evaluating use of AI in cancer diagnosis and prognosis using multi-omics data, radiomics, pathomics, and/or clinical and laboratory data. The following were excluded from this study: 1. duplicates, 2. review articles, 3. systematic reviews, 4. absence of AI implementation, 5. study aims that were not associated with our theme, 6. inappropriate data type, 7. studies that mislabeled LASSO-Cox method as machine learning, 8. studies with sample size of less than 100, 9. a study protocol, 10. a study that failed to specify the particular machine learning technique employed, and 11. animal studies.

## 3. Results

**Figure 2:**
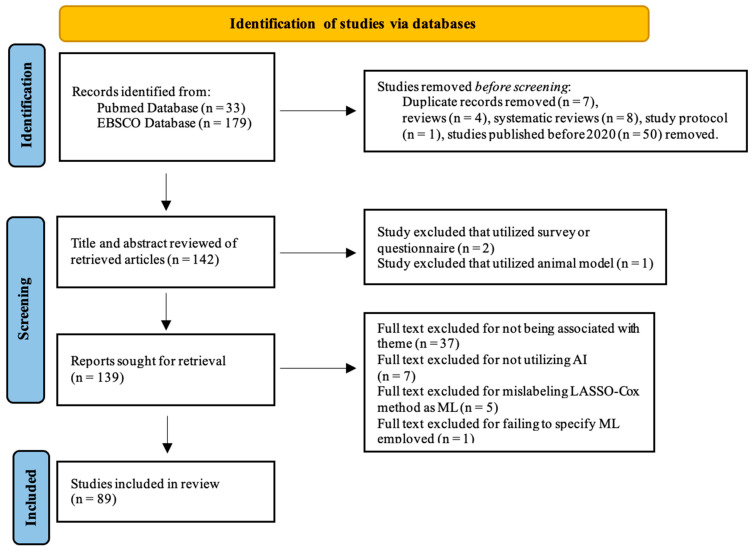
Flow diagram of the selection of studies to be included in the review.

**Figure 3:**
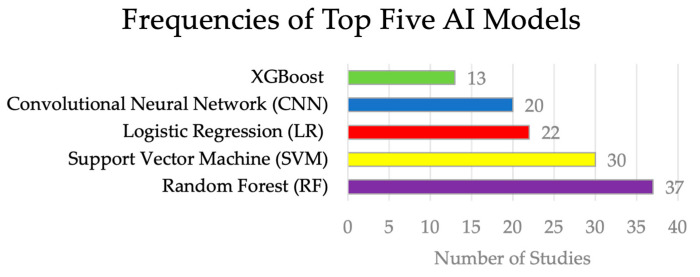
Frequencies of top five AI models.

In the analysis of 89 studies, we found a broad spectrum of AI applications within cancer research (Figure 2). There were two articles focusing on genomics data, twenty-one articles on transcriptomics data, three articles on epigenomics data, one article each on proteomics and metabolomics data, eight articles on multiomics data, thirty articles on radiomics data, three articles on pathomics data, and twenty articles on clinical data. No article on microbiomics data was found. Among these studies, 35 articles were pertinent to cancer diagnosis, while 54 articles were about cancer prognosis. Figure 3 shows a visual representation of the frequency of the top five AI models employed.

**Figure 4:**
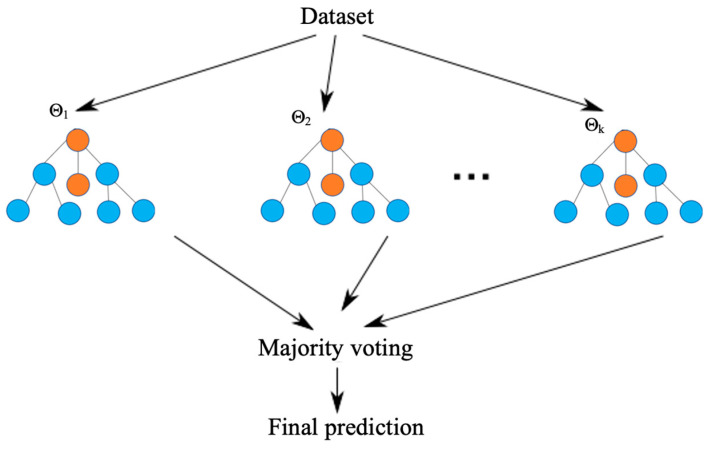
Schematic of Random Forest (RF); this image was adapted and modified from the following study [16]. The copyright of the image has been confirmed and verified.

The Random Forest (RF) method was the most prominently employed method. Studies that we reviewed with all data types except for pathomics did use RF. RF is a ML classifier composed of a collection of tree-structured classifiers {h(x, Θ_k_), k = 1, …} where the {Θ_k_} are identically distributed random vectors and each tree casts a unit vote for the most popular class from a dataset [11]. Each decision tree within RF is trained on Θ_k_, which is a random subset of the training data and features, as illustrated in Figure 4. During prediction, the output of each tree is aggregated to produce the final prediction. This integration of multiple decision trees serves to improve the accuracy and robustness of RF.

Support Vector Machine (SVM), Logistic Regression (LR), and XGBoost are legacy methods that are very well discussed and utilized in the literature. Here we refer the readers to the existing discussion [12,13,14]. However, since Deep Neural Network approaches such as Convolutional Neural Networks (CNNs) are considered state-of-the-art with the most impact, here we provide a brief discussion of Convolutional Neural Networks. It is also noteworthy that CNNs were the most popular method in radiomics and pathomics data analysis. A CNN is a type of DL model that uses convolutional operations to find important features in input data by overlapping and combining local areas [15]. This helps the network to recognize patterns even when they are not pre-labeled in the training data. The first step is to extract features from the input image. These features are then combined and reduced in size through pooling before being turned into the final network outputs. The last layers of the CNN connect all the neurons together and act as classifiers by sorting the input into different categories. Finally, the output layer gives the final classification or regression result, often using Softmax to calculate class probabilities.

For clarity and organization, the studies were categorized into eight sections based on the type of data utilized. Each section was further subdivided into two subsections focusing on cancer diagnosis and prognosis, respectively. Within these subsections, pertinent information from the articles was systematically collated into tables, including the title, author and year, study aim, modality of AI employed, and outcome or performance. Each table serves as a discrete subset under either cancer diagnosis or prognosis to facilitate efficient referencing and comparison. Articles under each table were then organized based on their study aim.

In evaluating the performance of AI models, it is important to understand several parameters, including accuracy, sensitivity, specificity, area under the curve (AUC), and concordance index (C-index). Accuracy measures the proximity of measurements to their true values. Sensitivity evaluates a model’s ability to predict true positives, while specificity assesses the model’s capacity to predict true negatives. AUC gives a comprehensive measure of performance across various classification thresholds, calculated as the area under the ROC curve. Then C-index, like the AUC, assesses the performance of prediction models, particularly in the context of survival analysis. A C-index closer to 1.0 indicates better predictive performance. In addition to the parameters, various AI algorithms or statistical methods were compared to evaluate the performance of AI.

### 3.1. Clinical Applications Based on Genomics

The following studies in Table 1 made notable contributions to the field of genomics by leveraging computational algorithms to predict key genetic patterns and treatment responses in cancer patients. Based on our search and exclusion criteria, there were only two papers on genomics data for various reasons (elaborated upon in the Discussion section). Because of the ability of modern AI to incorporate multimodal data, genomics data are often accompanied by other types of data, and therefore in this manuscript they are discussed in other sections. Typically, genomics data are combined with health records and other patient data. It is also possible (in theory) to combine genomics and some form of imaging. for instance, genomics data are often combined with radiology images or other -omics fields.

### 3.2. Clinical Applications Based on Transcriptomics

The following studies in Table 2 advanced the field of transcriptomics by employing machine learning (ML) and deep learning (DL) methods to analyze gene expression data and identify biomarkers associated with cancer. In terms of cancer prognosis, these studies employed ML methods to identify RNA signatures associated with various aspects of cancer prognosis and treatment response.

### 3.3. Clinical Applications Based on Epigenomics

The following studies in Table 3 contributed to epigenomics by employing various ML techniques to analyze epigenetic data and uncover important insights related to cancer prognosis and mutation detection.

### 3.4. Clinical Applications Based on Proteomics and Metabolomics

The following studies in Table 4 employed various ML techniques to analyze proteomics and metabolomics data.

### 3.5. Clinical Applications Based on Multiomics data

The following studies in Table 5 significantly advanced the field of multiomics by introducing innovative approaches to integrate diverse data types for cancer research. Multiomics data included genomics, transcriptomics, epigenomics, and proteomics. In terms of cancer prognosis, these studies leveraged various omics data and integrated them with clinical features to predict important outcomes in cancer.

### 3.6. Clinical Applications Based on Radiomics

In radiomics, these studies in Table 6 used ML and DL techniques for various tasks, including the classification of malignant versus benign tumors, gene expression prediction, and cancer invasion prediction. In terms of cancer prognosis, these studies achieved several advancements in predictive modeling and prognosis assessment for survival, metastasis prediction, and treatment complications.

### 3.7. Clinical Applications Based on Pathomics

In the field of pathomics, the following studies in Table 7 made notable contributions to cancer diagnosis and treatment response prediction by employing CNN models and were able to highlight the potential of pathomic analyses in personalized medicine and treatment optimization for cancer patients.

### 3.8. Clinical Applications Based on Clinical and Laboratory Data

In the field of clinical data analysis, the following studies in Table 8 showcased the integration of diverse data modalities for cancer prediction and classification by collectively highlighting the potential of integrating clinical and traditional medical data with ML approaches to enhance cancer diagnosis and prognostication. In terms of cancer prognosis, these studies made significant strides in utilizing ML methods for survival prediction, recurrence prediction, and treatment response assessment across various cancer types. These studies collectively demonstrate the effectiveness of ML approaches in leveraging clinical data to predict cancer prognosis, recurrence risk, and treatment outcomes, thus paving the way for personalized cancer management strategies.

## 4. Discussion

In this review, we presented various AI techniques that utilize multi-omics, radiomics, and pathomics, as well as clinical and laboratory data. While some studies focused solely on assessing AI’s performance using individual data types, a significant proportion incorporated the integration of diverse data types. This is because of the unique ability of modern ML techniques to integrate heterogenous modes of data to provide a more informed inference. Combining gene mutations with social and behavioral determinants of health will clearly address some of the long-standing challenges in precision and personalized medicine. It is only through a more holistic evaluation of a patient that the most accurate diagnosis and prognosis can be determined. This is one of the primary reasons why most studies incorporate multiple data modalities in their AI implementations. In addition, some forms of data are a poor fit for analysis with AI and therefore are relatively less frequently explored. For instance, it is difficult to formulate an AI/ML-based approach to the analysis of purely genomic data. The most comprehensive approach to genomic analysis would require an AI capable of accepting four billion inputs (the human genome size) to provide an inference. There are many practical limitations in the development of such a network, including computation time, memory capacity of existing computers, and the curse of high dimensionality of the data to name a few. A more pragmatic approach is to limit the analysis to a limited number of genes and loci as relevant indicators that are selected based on other data such as differentially expressed genes, gene methylations, or gene pathway analysis. Studies that were purportedly limited to a single data type integrated demographic information into their AI models. This integrated approach is advantageous given the complexity of cancer as a biological phenomenon, consequently bolstering diagnostic and prognostic capabilities. With cancer being one of the leading causes of death, improving diagnosis and prognosis is an area of medicine that has caught the attention of many physicians and researchers.

In this manuscript, we have aimed to maintain an agnostic position in reporting a summary of the published work and to remain neutral with regards to recommending the best approach and promoting one work over another. In fact, such a discrimination would be very difficult to accomplish since the most successful approach will vary based on the data modality. For instance, convolutional neural networks are generally recognized to be the most suitable approach in analysis and segmentation of images. On the other hand, recurrent neural networks such as LSTM would be most appropriate in application to temporal (potentially longitudinal) analysis such as demand forecasting (e.g., hospital resource use, medication dosage, etc.). In domains where the interpretability of the mechanism by which machines are operating is of critical importance, decision trees or Random Forests may be the most appropriate approaches. Another complication in making naïve comparisons between two methods is the uniformity of the data or other evaluation conditions. For example, two image segmentation methods applied to CTA scans of the abdominal cavity may produce results of 80% and 90%. While it is simple to conclude the former method’s superiority over the latter, the question remains whether the quality of the data collected between the two experiments was comparable or not. How about the sample size or diversity of the data? Many factors need to be normalized across all studies to draw a meaningful comparison between different approaches. This is the primary impetus in the need for normalization and standardization of data within the framework of AI/ML evaluation.

We noted that diverse datasets often comprise many features. Some studies have noted overfitting in their models due to the utilization of a larger number of features relative to a smaller sample size [66,96]. This issue is commonly referred to as the ‘n << P problem,’ where ‘n‘ represents the sample size and ‘P‘ denotes the number of features [106]. Dealing with many features in data can pose challenges when employing AI models, particularly in the context of high dimensionality. One significant challenge associated with high dimensionality is the increased sparsity of data, where information becomes thinly distributed across the feature space. Imagine each piece of data as a dot on a graph. As we add more and more features, the space where these dots exist gets bigger and bigger, making the dots more spread out, or “sparse”. Consequently, making accurate predictions becomes challenging unless a substantial number of data points are available. This difficulty is particularly pronounced when analyzing medical data since it often exhibits considerable variation. Hence, researchers take steps to maximize the number of available samples while minimizing the number of features. We observed that many studies have adopted various feature selection and extraction techniques to address this challenge.

Feature selection and extraction can be accomplished by human experts or with computational algorithms. ML methods such as SVM and RF, along with statistical methods including the LASSO–Cox model, were frequently employed for feature selection. Autoencoder, a type of ML algorithm, was a popular method to integrate multi-omics ML data. DL methods were applied more extensively in radiomics data analysis for feature selection and extraction. This preference for DL, particularly CNNs, stems from their efficiency in handling large volumes of data compared to traditional ML or statistical methods. Additionally, CNNs automate the process of feature extraction and classification by identifying patterns and extracting features from images. A limitation of DL lies in its ‘black box problem’, where it fails to offer interpretations to justify model findings or provide additional clinical insights. Despite this challenge, efforts have been made to demonstrate the importance of features extracted by CNNs. For instance, researchers like Fujima et al. attempted to validate significant radiomic features extracted using CNN through statistical analysis [73]. Unlike DL methods, statistical methods such as the Cox Proportional Hazards (PH) model offer interpretable outcome values. Shapley values derived from the SHapley Additive exPlanations (SHAP) algorithm can interpret outcomes derived from ML methods [86]. Shapley values offer insights into the contributions of features towards specific outcomes.

Another approach to address the ‘n << P problem’ involves increasing the sample size. Many studies have leveraged data from publicly available datasets such as The Cancer Genome Atlas (TCGA). However, excessive reliance on TCGA data may introduce bias towards the -omics data types present in the TCGA dataset, potentially leading to the overfitting of models and resulting in bias and misrepresentation of the outcome. Therefore, initiatives aimed at providing large-scale, multi-modal datasets to the research community are necessary. Moreover, studies that increased the number of samples encountered challenges related to imbalanced data. To mitigate this issue, Meng et al. and Hu et al. employed the Synthetic Minority Over-sampling Technique (SMOTE) algorithm, which replicates minority class samples.

Using AI to diagnose cancer or make prognoses for cancer patients involves several nuanced ethical considerations. Ensuring accuracy and reliability is very important, as errors can lead to significant harm, such as unnecessary treatments or missed opportunities for early intervention. AI’s decision-making process needs to be transparent to maintain trust between patients and healthcare providers. Moreover, patient privacy and data security must be safeguarded, given the highly sensitive nature of medical information. AI should augment, not replace, human judgment, ensuring that medical professionals remain central to providing comprehensive and compassionate patient care.

## 5. Conclusions

In this review, we provide a comprehensive synopsis of some of the most promising AI utilizations and discuss the limitations associated with each method. AI can significantly improve the cost-effectiveness of cancer diagnosis and treatment by enhancing accuracy, personalizing care, and improving operational efficiencies. Interdisciplinary collaboration is essential in advancing AI applications in oncology. Ethicists, legal experts, and policymakers should be included in the interdisciplinary teams to navigate the complex landscape of AI deployment in oncology. By bringing together diverse expertise from various fields, these collaborations ensure the development of robust, clinically relevant, and ethically sound AI tools that can significantly improve cancer diagnosis and treatment. Challenges persist in achieving feature reproducibility and in ensuring model interpretability.

Overall, most AI models examined in this study were focused on tasks such as classification, clustering, or regression. These models have demonstrated promising outcomes and performance; however, they are not currently suitable for use in clinical settings. This limitation arises from various contributing factors, including lack of standardization of the data, normalization procedures, and evaluation of models by multiple independent investigators. While the list continues to grow as examined further, the primary root of all existing limitations is based on the private nature of medical data. For instance, evaluation of a model by an independent entity may face obvious challenges regarding IRB data sharing requirements. Once the primary impediment of data sharing is resolved, retrospective evaluation by multiple investigators can add confidence to the use of a trained network, which will lead to its translational deployment in clinical settings. Thus, robust prospective studies are necessary to guarantee the safety and efficacy of AI models. Furthermore, concerted efforts to enhance algorithm interpretability and comprehend human–algorithm interactions will be important for future adoption and safety.

## Figures and Tables

**Table 1:** Genomics-based prediction of cancer prognosis.

Outcome/Performance	Sample Size	Modality of AI	Study Aim and Cancer Type	Author, Year
Genomics-based prediction of prognostic biomarker				
A total of 8 (14.8%), 10 (16.9%), 17 (18.7%), and 43 (59.7%) cases were predicted to exhibit the *MYC*-trans, *BCL2*-trans, *BCL6*-trans, and MC signatures. Neither external nor independent validation was performed.	342 patients	RF	To identify overlapping genetic patterns in DLBCL patients	Zhang et al., 2020 [17]
Genomics-based prediction of treatment responses				
Mean root square error (0.1587) of ANN-SCGP was lowest among other traditional MLs, including RF, SVM, and ANN. Mean root square error assesses the average difference between the predicted values generated by a model and actual values. Neither external nor independent validation was performed.	1101 patients	ANN with Selective Connection based on Gene Patterns (ANN-SCGP) RF, support vector machine (SVM), ANN, DeepSurv	To predict treatment response to radiotherapy based on gene patterns	Zeng et al., 2022 [18].

**Table 2:** Transcriptomics-based prediction of cancer diagnosis and prognosis.

Outcome/Performance	Sample Size	Modality of AI	Study Aim and Cancer Type	Author, Year
Transcriptomics-based cancer detection				
An 11-gene panel was validated with GB for accuracy in distinguishing NSCLC cases from healthy controls. Among the three classifiers, GBM offered the highest AUC = 0.97. Neither external nor independent validation was performed.	273 samples	Gradient Boosting Machines (GBM) and RF	To discriminate between non-metastatic NSCLC cases and healthy samples using 11 platelet genes	Goswami et al., 2020 [19].
The model trained with 19 primary cancer types achieved the highest performance with 89.67%, 87.32%, and 84.59% accuracy for 6-, 8-, and 10-way predictions in test samples. Neither external nor independent validation was performed.	11,105 samples	Siamese convolutional neural network (SCNN)	To predict cancer types for primary and metastatic tumors from gene expression data	Mostavi et al., 2021 [20].
Transcriptomics-based classification of malignant vs. benign tumors				
Mean root square error (0.1587) of ANN-SCGP was lowest among other traditional ML algorithms, including RF, SVM, and ANN. Mean root square error assesses the average difference between the predicted values generated by a model and actual values. External validation performed with their tissue microarray data.	2214 samples	ANN with Selective Connection based on Gene Patterns (ANN-SCGP) RF, support vector machine (SVM), ANN, DeepSurv	To predict treatment response to radiotherapy based on gene patterns	Carrillo-Perez et al., 2021 [21].
Transcriptomics-based survival prediction				
The AUC values of four survival groups were all above 90%. The patient groups predicted by the SVM model demonstrated comparable survival outcomes to those clustered by the K-means algorithm. Neither external nor independent validation was performed.	542 miRNAs and 312 samples.	Combination of K-means clustering and SVM	To evaluate a microRNA-based machine learning survival prediction model	Ding et al., 2021 [22].
The stemness subtype classifier by RF showed good performance in the classification with an AUC of 0.956, and the sensitivity, specificity, and accuracy were 86.15%, 91.03% and 88.9%. External validation was performed with total of 169 samples from three gene set enrichment analyses.	478 lung cancer tissues and 50 normal samples.	RF	To predict transcriptional stemness indices of lung cancer from RNA expression data	Lai et al., 2023 [23].
RF approach outperformed traditional prognostic variables like disease stage and cell of origin (COO) in predictive accuracy for DLBCL patients. Independent validation was performed with gene set enrichment analysis from 69 patients.	420 patients	RF	To evaluate new machine learning-based models of survival prediction using transcriptomic and clinical data	Mosquera Orgueira et al., 2020 [24].
Transcriptomics-based recurrence prediction				
SVM-REF and Random Forest analyses selected 66 and 30 lncRNA prognostic signatures, respectively. Neither external nor independent validation was performed.	314 patients	RF and Support Vector Machine Recursive Feature Elimination (SVM-RFE)	To evaluate a lncRNA-based signature for predicting early HCC recurrence	Fu et al., 2023 [25].
Prediction of breast cancer recurrence with XGBoost performed better with mRNA data (AUC = 0.74) alone compared to mutation alone (AUC = 0.62). Neither external nor independent validation was performed.	2000 samples	XGBoost	To evaluate prognostic utility of genomic mutations to that of gene expression using breast cancer data	Ravkin et al., 2020 [26].
Transcriptomics-based prediction of risk stratification				
SVMR achieved the best classification performance (accuracy = 0.923, sensitivity = 0.927, specificity = 0.919) compared to other classifiers. Independent validation was performed.	325 samples	Support vector machine with radial kernel (SVMR)	To identify a novel miRNA signature related to tumor stage and prognosis of clear cell renal cell carcinoma patients	Dessie et al., 2021 [27].
Combining differentially spliced and expression levels of RNA yielded the most performant RF classifier compared to splicing signature only or expression levels only. Neither external nor independent validation was performed.	Sample size was not specified	RF	To subclassify highly aggressive breast cancers with transcriptomics analysis of alternative splicing events	Villemin et al., 2021 [28].
SWT-CNN outperformed other machine learning algorithms including support vector machine (SVM) and logistic regression (LR). SWT-CNN performed comparably with RF in predicting tumor stages. Independent validation was performed.	34,534 unique protein-coding genes and lncRNA genes	Combination of a convolutional neural network with stationary wavelet transform (SWT-CNN)	To stratify the prognostic risk for cancer patients by using SWT-CNN	Zhao et al., 2020 [29].
Transcriptomics-based prediction of prognostic biomarker				
RF exhibited the highest Area Under the Curve (AUC) across all datasets, while SVM demonstrated the highest sensitivity and specificity. Neither external nor independent validation was performed.	Sample size was not specified	RF, KNN, SVM, naïve Bayes (NB), and neural networks (NNET) for feature extraction	To identify transcript biomarkers that could help in early prognosis for HCC	Gupta et al., 2021 [30].
The top five significant molecules pinpointed by each machine learning algorithm revealed a single intersecting molecule which is SFN. Independent validation was performed with 30% of sample.	179 patients	LR, SVM, artificial neural network (ANN), RF, and XGBoost	To identify key prognostic molecule with multiple ML algorithms	Li et al., 2021 [31].
RF ranked top 10 important master genes for two prognostic groups, including *CCNA2, CBX7*, *TMEM48*, *SPC25*, *GAPDH*, *WDHD1*, *PSMD2*, *ERO1L*, *DDX52*, and *ARNTL2*. Neither external nor independent validation was performed.	515 patients	RF	To identify the key prognosis impacting genes and relevant subtypes for lung adenocarcinoma	Lv and Lei., 2020 [32].
A machine-learning-based approach identified C5AR1/SYT5 and MSR1/SLC32A1 signatures which discriminated NL IDH-WT gliomas with high sensitivity and specificity in various glioma expression datasets. Neither external nor independent validation was performed.	Sample size was not specified	K-nearest neighbor (KNN)	To characterize novel biomarkers in gliomas	Nguyen et al., 2020 [33].
SVM-RFE yielded 72 prognostic features with classification accuracy of 0.934. External validation was performed with total of 764 samples from three gene set enrichment analyses.	365 samples	SVM-RFE	To evaluate the association between immune infiltration and prognosis in ovarian cancer	Yan et al., 2020 [34].
The intersection of the top 10 feature lncRNAs obtained from both the XGBoost and Boruta algorithms resulted in eight intersecting lncRNAs. External validation was performed with International Cancer Genome Consortium dataset.	531 cancer samples and 72 normal samples	XGBoost and Boruta algorithm	To identify and explore prognostic biomarkers associated with clear cell renal cell carcinoma	Zhong et al., 2023 [35].
Transcriptomics-based prediction of laterality of cancer				
SVM-RBF classified the different locations by the highest accuracy of 99%. RF classified with high accuracy. NB was not satisfactory. Neither external nor independent validation was performed.	450 samples	NB, SVM-RBF, and RF	To identify biomarkers which are associated with specific tumor locations	Hamzeh et al., 2020 [36]
Transcriptomics-based prediction of treatment responses				
RF yielded best results with mean accuracy of 84.1% for 5-FU and 82.3% for GCB. Independent validation was performed.	Sample size was not specified	RF, SVM, LR	To predict treatment response of multiple cancer types to 5-Fluorouracil and Gemcitabine	Clayton et al., 2020 [37].
Cluster 2 exhibited a notably poorer prognosis compared to Cluster 1. Neither external nor independent validation was performed.	Sample size was not specified	K means clustering	To examine relationships between the effects of platinum-containing drugs and those of metabolic genes and FAK activity in advanced ovarian high-grade serous carcinoma	Sato et al. 2022 [38].
KNN-derived AUC of 0.72. This model performed better than previously published pan-cancer predictive models for immunotherapy efficacy. External and independent validation was performed.	Sample size was not specified	KNN	To predict survival and immunotherapy response with transcriptomic marker from tumor endothelial cells	Wu et al., 2023 [39].

**Table 3:** Epigenomics-based prediction of cancer diagnosis and prognosis.

Outcome/Performance	Sample Size	Modality of AI	Study Aim and Cancer Type	Author, Year
Epigenomics-based classification of malignant vs. benign tumors				
SETRED with SVM base learner performed the best, with mean accuracy above 0.95 and AUCs for methylation class and family prediction (AUC = 0.73 and 0.94, respectively). The NN model exhibited notably higher balanced accuracy (92.9% and 97.5%) compared to the RF classifier (70.9% and 72.3%). Independent validation was performed with 30% of sample.	2801 samples	Eleven semi-supervised learning models based on SVM, decision tree, and one nearest neighbor; two supervised classification models: RF and NN	To explore utility of semi-supervised models in methylation data	Tran et al., 2022 [40].
Epigenomics-based classification of tumor staging				
Precisions for groups A–D were 0.931, 0.833, 0.577, and 0.414. Neither external nor independent validation was performed.	493 samples	RF	To classify neuroblastoma staging with epigenomics data	Sugino et al., 2022 [41].
Epigenomics-based prediction of biomarker for cancer prognosis				
The model yielded a sensitivity of 0.94, specificity of 0.82, and a false negative rate of 0.06. External validation was performed.	Sample size was not specified	Binomial logistic regression	To develop and validate a 3-CpG methylation signature to predict *SETD2* mutation status	Javaid et al., 2023 [42].

**Table 4:** Proteomics- and metabolomics-based prediction of cancer diagnosis and prognosis.

Outcome/Performance	Sample Size	Modality of AI	Study Aim and Cancer Type	Author, Year
Proteomics-based prediction of diagnostic biomarker				
A diagnostic model (RF) incorporating seven factors (CLU, CA19-9, IBIL, GGT, LDL-C, TG, and TBA), showed a high diagnostic utility with AUC: 0.947, sensitivity: 90.3%, and specificity: 84.9%. External validation was performed with 259 patients.	644 patients	RF	To evaluate diagnostic performance of proteomic biomarker for cholangiocarcinoma	Gao et al., 2023 [43].
Metabolimics-based cancer prediction				
The XGBoost model showed the best predictive power (AUC = 0.81, accuracy = 75.29%, sensitivity = 74%). Neither external nor independent validation was performed.	478 patients	XGBoost, SVM, KNN, RF	To predict lung cancer with metabolic data	Guan et al., 2023 [44].

**Table 5:** Cancer diagnosis and prognosis based on multiomics data.

Outcome/Performance	Sample Size	Modality of AI	Study Aim and Cancer Type	Author, Year
Cancer prediction based on multiomics data				
The AUC of LGDLDA was 0.880, which was 0.034, 0.088, 0.053, and 0.208 higher than that of IDHI-MIRW, NCPLDA, LncDisAP, and NCPHLDA, respectively. Neither external nor independent validation was performed.	Sample size was not specified	LncRNA-Gene-Disease association networks based LncRNA-Disease Association prediction (LGDLDA); base model is a neural network	To identify cancer-related lncRNAs	Yuan et al., 2021 [45].
Subclassification of malignant tumors based on multiomics data				
Mean root square error (0.1587) of ANN-SCGP was lowest among other traditional ML algorithms, including RF, SVM, and ANN. Mean root square error assesses the average difference between the predicted values generated by a model and actual values. Neither external nor independent validation was performed.	1059 samples	moBRCA-net; base model is a neural network	To evaluate moBRCA-net	Choi and Chae, 2023 [46].
Survival prediction based on multiomics data				
Survival prediction: accuracy of 94% and AUC of 0.98 Drug response prediction: AUC of 0.83 and 0.78 for Docitaxel and Gemcitabine External validation was performed with TCGA dataset.	Sample size was not specified	Neural network-based classifier	To predict survival and drug response for breast cancer patients	Malik et al., 2021 [47].
Autoencoder outperformed two statistical methods with C-index of 0.92 (PCA and iCluster). External validation was performed with Chinese Glioma Genome Atlas dataset.	563 samples	Combination of Autoencoder and SVM	To identify survival subtype of glioma with RNA expression and DNA methylation data	Tian et al., 2022 [48].
DNA methylation and miRNA expression resulted in best performance with C-index of 0.641. Independent validation was performed with 10,000 samples.	60,000 samples	Concatenation autoencoder (ConcatAE) and CrossAE	To predict breast cancer survival by integrating multi-omics data	Tong et al., 2020 [49].
Multiomics-based prediction of prognostic biomarker				
A total of 75 mRNAs were identified as prognostic in TCGA cohort. A total of 29 mRNAs were identified as prognostic in LIRI-JP dataset. Independent validation was performed with the Liver Cancer, Riken Japan (LIRI-JP) HCC dataset	352 patients	Autoencoder	To identify biomarkers that distinguish prognostic subgroups in liver cancer	Owens et al., 2021 [50].
Multiomics-based prediction of laterality of cancer				
The classification model derived from the 17 gene expressions resulted in an AUC of 0.96. Neither external nor independent validation was performed.	283 patients	XGBoost	To identify gene mutation and expression patterns between left-sided and right-sided colon cancer	Jiang et al., 2020 [51].
The accuracies of the RF models were 90%, 70%, and 87% with corresponding area under the curve (AUC) values of 0.9, 0.76, and 0.89 for the human genomic, microbial, and combined feature sets, respectively. Independent validation was performed with 30 samples.	308 samples	RF	To predict sidedness of colon cancer	Kolisnik et al., 2023 [52].

**Table 6:** Radiomics-based prediction of cancer diagnosis and prognosis.

Outcome/Performance	Sample Size	Modality of AI	Study Aim and Cancer Type	Author, Year
Radiomics-based classification of malignant vs. benign tumors				
Xception AUC: 0.970 DenseNet169 AUC: 0.959 Both DL algorithms outperformed radiologists (*p* < 0.05). Independent validation was performed with 20% of sample.	546 samples	Xception DenseNet169 DenseNet121 NASNetLarge ResNet101v2	To evaluate diagnostic performance of DL algorithms in distinguishing benign vs. malignant thyroid calcified nodules	Chen et al., 2023 [53].
Fusion model achieved AUC of 0.916 for SCA diagnosis and AUC of 0.973 for MCA and IPMN diagnosis. Neither external nor independent validation was performed.	193 patients	Fused model: Based on LR and SVM	To evaluate diagnostic models based on radiomics and deep learning algorithms to differentiate three types of pancreatic cystic neoplasms	Liang et al., 2022 [54].
A total of 180 tumor texture features were extracted from enhanced CT and unenhanced CT. Neither external nor independent validation was performed.	188 patients	AK software (Artificial Intelligence Kit V3.0.0.R) by GE Healthcare	To diagnose anterior mediastinal cysts vs. thymomas with radiomic features	Liu et al., 2020 [55].
Mean accuracy of 93.25%, a sensitivity of 89.22%, a specificity of 95.82%, and AUC of 0.9629. Neither external nor independent validation was performed.	Sample size was not specified	RGB: combination of five CNNs and one GCN	To evaluate RGB model classification of benign vs. malignant lung nodules	Ma et al., 2023 [56].
The DLR model achieved an AUC of 0.986, 0.978, 0.967, and 0.953 in the training, internal validation, and external validation.	558 patients	DLR model: based on ResNet50	To evaluate role of deep learning radiomics on contrast-enhanced US in distinguishing pancreatic adenocarcinoma vs. chronic pancreatitis	Tong et al., 2022 [57].
CNN model with clinical features achieved the highest AUC at 0.819. Neither external nor independent validation was performed.	720 samples	CNN and RF	To distinguish benign vs. malignant lung nodules in chest CT	Zhang et al., 2022 [58].
The model established by the LR method had the best performance, and the AUC values in the training group and test group were 0.840 and 0.960. The AUC of the combined model was 0.940, 0.990, and 0.960 in the training group, test group, and external validation group.	177 patients	Radiomics model: RF, SVM, and LR; combined model: LR	To differentiate pulmonary mucinous adenocarcinoma from tuberculoma based on features from CT images and clinical features	Zhang et al., 2023 [59].
The accuracy of the test set was 0.84. A total of 96 images from the test set without data augmentation were analyzed and the accuracy was 0.89. Both external and independent validation were performed.	477 patients	PB-LNet: Based on ResNext50 and Bidirectional LSTM (BiLSTM)	To classify CT images of lung nodules into six categories based on pathological subtypes	Zhang et al., 2023 [60].
AUCs for lymphoma ranged from 0.670 to 0.936 in three testing sets. AUCs for metastatic carcinoma ranged from 0.804 to 0.855 in three testing sets. Both external and independent validation were performed.	763 patients	ResNet50	To accurately diagnose unexplained cervical lymphadenopathy with ultrasound	Zhu et al., 2022 [61].
Radiomics-based prediction of gene expression in malignant tumors				
AUCs of the clinical model (LR) in the testing, internal validation, and external validation sets were 0.794, 0.711, and 0.75. AUCs of the deep models and joint models ranged from 0.939 to 0.993.	229 patients	One ML model: LR; Three DL models:	To predict Ki67 expression in prostate cancer with MRI radiomics	Deng et al., 2023 [62].
The predictive performance of the DLRS-Resnet model was inferior to that of the Nomogram-Resnet model (*p* < 0.01). Both external and independent validation were performed.		DLRS-Resnet, DLRS-Inception, and DLRS-Densenet Three joint models: Nomogram-Resnet, Nomogram-Inception, and Nomogram-Densenet		
ResNet model in the axial direction achieved the higher AUC of 0.90 in the testing cohort than coronal or sagittal directions. The AUC of radiomics model (RF) in testing cohorts was 0.818. Independent validation was performed.	156 patients	ResNet and RF	To predict *KRAS* mutation in colorectal cancer with CT radiomics	He et al., 2020 [63].
Radiomics-based prediction of cancer invasion				
Radiomics model (LR) performed best in training and external dataset. Combined model (LR) performed best in the testing set. Independent validation was performed with 30% of sample.	86 patients	LR	To predict lymphovascular invasion status in cervical cancer	Li et al., 2021 [64].
Three SVM-based prediction models demonstrated relatively high efficacy in identifying LVI of breast cancer, with AUCs of 79.00%, 80.00%, and 79.40% and an accuracy of 71.00%, 80.00%, and 75.00% in the validation cohort for AP, SP, and CP plane image. Fusion model achieved the highest AUC of 87.90% and an accuracy of 85.00% in the validation cohort. Independent validation was performed with 30% of sample.	434 patients	SVM	To predict the lymphovascular invasion status in breast cancer	Li et al., 2023 [65].
FNN performed the best, with CMP demonstrating the highest AUC at 0.81. Neither external nor independent validation was performed.	126 patients	SVM-RBF, KNN, LR, linear discriminant analysis (LDA), forward neural network (FNN)	To predict capsule invasion in renal cell carcinoma	Yang et al., 2022 [66].
The six models showed a certain value of radiomics, with AUCs from 0.642 to 0.701. LR demonstrated the best performance. Independent validation was performed with 30% of sample.	153 patients	KNN, LR, decision tree, linear SVM, Gaussian SVM, polynomial SVM	To predict extrathyroidal extension (ETE) in papillary thyroid cancer (PTC) patients	Yu et al., 2022 [67].
XGBoost model demonstrated the best performance in both training and testing set with AUCs of 0.917 and 0.874. Neither external nor independent validation was performed.	203 samples	LR, SVM, XGBoost	To predict histological invasiveness of sub-centimeter subsolid pulmonary nodules	Zhang et al., 2023 [68].
Radiomics-based survival prediction				
EN and RF achieved top prognostication performances of AUC = 0.795 and AUC = 0.811. RF prognostication slightly outperformed the EN for the complete and radiochemotherapy cohort. Independent validation was performed.	157 patients	Elastic Net (EN); RF	To predict survival of patients with squamous cell carcinoma of the head and neck with CT radiomics	Bernatz et al., 2023 [69].
The overall prediction accuracy for 3-year survival status in training and validation cohort was 92.50% and 85.71%, and the AUCs were 0.965 and 0.869. Independent validation was performed with 33% of sample.	298 patients	SVM	To predict survival of unresectable lung cancer patients with CT radiomics	Chen et al., 2022 [70].
RF models built with clinical, CT, and PET features outperformed other models with solely clinical, PET, or CT features with C-indices 0.780 and 0.820 in training and testing set. Independent validation was performed.	196 patients	RF	To predict survival of colorectal cancer patients with ^8^F-FDG PET/CT radiomic features	Lv et al., 2022 [71].
For 2- and 5-year survival predictions, ResNet 50 achieved the best performance for 2D PET images, while ResNet 34 achieved the best performance for 3D PET images. ResNet 34 demonstrated the best performance with a C-index of 0.749. Neither external nor independent validation was performed.	2687 patients	ResNet50 for 2D PET images ResNet3D34 for 3D PET images	To predict survival of non-small cell lung cancer patients with PET radiomics	Oh et al., 2023 [72].
Radiomics-based metastasis prediction				
The patient demographic model resulted in accuracies of 67.31% and 73.08% and AUCs of 0.706 and 0.773 for training and testing cohorts. The radiomics-derived model resulted in accuracies of 81.09% and 79.49% and AUCs of 0.882 and 0.825 for training and testing cohorts. Neither external nor independent validation was performed.	390 patients	SVM	To predict lymph node metastasis with pre-op CT	Eresen et al., 2020 [73].
MNB outperformed other ML algorithms with AUC, specificity, and accuracy on the testing set of 0.745, 0.900, and 0.778. Neither external nor independent validation was performed.	180 patients	XGBoost, LR, multinomial naïve Bayes (MNB), SVM, decision tree, RF, gradient boosting decision tree (GBDT)	To predict lymph node metastasis in cervical cancer with MRI radiomics	Liu et al., 2023 [74].
XGBoost outperformed other MLs with AUC of 0.98, sensitivity of 0.75, and specificity of 0.94. Independent validation was performed.	100 patients	Ada boosting (ADA), bagging classifier (BAGC), Bernoulli naïve Bayes (BNB), decision tree, Gaussian naïve Bayes (GNB), KNN, RF, stochastic gradient descent (SGD), SVM, and XGBoost	To predict lymph node metastasis in extrahepatic cholangiocarcinoma	Tang et al., 2021 [75].
LR demonstrated the best performance, achieving an AUC of 0.754. Independent validation was performed with 30% of sample.	299 patients	SVM, KNN, RF, and LR	To predict distant metastasis in esophageal cancer	Zhu et al., 2022 [76].
Radiomics-based prediction of treatment responses				
The axial and coronal combination model in ResNet (AUC = 0.85) demonstrated the best performance. Independent validation was performed.	154 patients	AlexNet, GoogLeNet Inception v3, and ResNet-101	To predict treatment outcomes in oropharyngeal squamous cell carcinoma by DL algorithms	Fujima et al., 2021 [77].
The average accuracy of C-SVM, R-SVM, and C-R SVM were 0.712, 0.792, and 0.844, respectively, while the average AUC values were 0.775, 0.804, and 0.877. Independent validation was performed with 30% of sample.	106 patients	SVM	To predict prognosis of downstaging treatment in hepatocellular carcinoma	Wang et al., 2023 [78].
DLRPM exhibited superior prediction performance compared to single-scale prediction models, achieving an AUC of 0.927 in the validation set. Independent validation was performed.	211 patients	DLRPM: based on SVM	To predict responses to chemotherapy in breast cancer patients	Zhang et al., 2023 [79].
Radiomics-based prediction of treatment complications				
Combined model (RF) of radiation dose and radiomics resulted in best performance with AUC of 0.9993 and 0.9000 in training and testing set. Neither external nor independent validation was performed.	140 patients	ResNet50 for feature extraction RF for classification	To predict radiation pneumonitis after radiotherapy	Huang et al., 2022 [80].
RF achieved AUC range of 0.713 to 0.756. Both external and independent validation were performed.	761 patients	Linear SVM for feature extraction RF for classification	To predict post-radiation nasopharyngeal necrosis after radiotherapy	Liu et al., 2023 [81].
The radiomic models (N1, N2, N3) with longitudinal MRI yielded AUCs of 0.872, 0.836, and 0.780 for RTLI prediction. Independent validation was performed.	242 patients	RF	To predict radiation-induced brain injury after radiotherapy	Zhang et al., 2020 [82].

**Table 7:** Pathomics-based prediction of cancer diagnosis and prognosis.

Outcome/Performance	Sample Size	Modality of AI	Study Aim and Cancer Type	Author, Year
Pathomics-based prediction of cancer diagnosis				
Google Inception V3 yielded an average AUC of 98.06%. Both external and independent validation were performed.	14,234 samples	Google Inception V3	To diagnose colorectal cancer with DL on weakly-labeled WSIs	Wang et al., 2021 [83].
Pathomics-based classification of malignant vs. benign tumors				
Richer fusion network outperformed other models in the literature with an average accuracy of 92.9%. Neither external nor independent validation was performed.	3764 samples	Richer fusion network: based on Sparse denoising autoencoder and VGG16	To classify benign vs. malignant breast lesions with WSIs and EMR	Yan et al., 2021 [84].
Pathomics-based prediction of treatment responses				
VGGNet had the best predictive ability and was utilized as a backbone model to identify transcriptomic subtypes and predict therapy responses. Neither external nor independent validation was performed.	587 patients	AlexNet, GoogLeNet, and VGGNet	To evaluate a CNN model that diagnoses ovarian cancer and predicts treatment response	Yu et al., 2020 [85].

**Table 8:** Cancer diagnosis and prognosis based on clinical and laboratory data.

Outcome/Performance	Sample Size	Modality of AI	Study Aim and Cancer Type	Author, Year
Cancer prediction based on clinical and laboratory data				
CyPath resulted in AUC of 0.89, sensitivity of 82.1%, and sensitivity of 87.7% for test set and AUC of 0.94 for test set. Independent validation was performed with 32 new patients.	150 patients	CyPath Lung: based on LR	To detect lung cancer in sputum with ML	Lemieux et al., 2023 [86].
XGboost generated the highest AUC value of models, which were 0.915, 0.9529, 0.9557, and 0.9614 for diagnosing ASCUS higher, ASC-H higher, LSIL higher, and HSIL higher staged cervical lesions, indicating the acceptable accuracy of the selected diagnostic model. Independent validation was performed with 20% of sample.	48,565 patients	LR for feature selection; Six ML algorithms for classification: decision tree, XGBoost, RF, SVM, LR, and neural net	To predict cervical cancer with HPV screening dataset	Meng et al., 2022 [87].
AutoML had the highest AUC of 0.807 of four ML algorithms. AutoML had encouraging discriminative power with AUCs of 0.820 in the validation cohort and 0.807 and 0.850 in the two prospective test cohorts. Both external and independent validation were performed.	4747 patients	RF for feature selection; AutoML, LR, RF, and XGBoost for model establishment	To diagnose prostate cancer with clinical data	Zhang et al., 2023 [88].
RF model incorporating selected features exhibited excellent performance in predicting HCC events occurring within 1 year, achieving an AUC of 0.9507. Predictions for the 2-year and 3-year time frames also yielded favorable results, with AUCs of 0.8767 and 0.8307, respectively. Independent validation was performed with 30% of sample.	400 patients	RF	To predict risk of hepatocellular carcinoma in patients with hepatitis C cirrhosis	Zou et al., 2023 [89].
Classification of malignant vs. benign tumors based on clinical and laboratory data				
The XGBoost model provided better performance (AUC of 0.82) compared with free-to-total PSA ratio (AUC of 0.75), total PSA (AUC of 0.68) and free PSA (AUC of 0.61). Independent validation was performed with 30% of sample.	1915 patients	XGBoost	To distinguish benign prostate hyperplasia from prostate cancer using ML	Chen et al., 2023 [90].
Xception CNN showed AUROCs of 0.8741, 0.9199, and 0.8363 for the detection of myeloblasts, promyelocytes, and Auer rods. ENNs resulted in AUCs of 0.8575 and 0.9585 in distinguishing between APL and non-APL AML as well as APL and healthy donors. Neither external nor independent validation was performed.	1335 samples	XceptionCNN to label cell border Binary ensemble neural nets (ENNs) for classification	To predict acute promyelocytic leukemia from bone marrow smear images	Eckardt et al., 2022 [91].
Xy-SkinNet achieved a 64.75% accuracy rate for its top-ranked diagnosis, surpassing the average performance of dermatologists, which stood at 62.13%. Neither external nor independent validation was performed. Instead, this study conducted a contrast experiment with 31 dermatologists.	5660 samples	Xy-SkinNet: based on ResNet and Fast R-CNN	To classify six common skin diseases with AI	Huang et al., 2021 [92].
Tumor grading based on clinical and laboratory data				
Incorporating additional non-image information such as cytology and HPV status improved CAIADS’ diagnostic performance, with an AUC of 0.712 for LSIL and 0.829 for HSIL and cancer. CAIADS surpassed the diagnostic performance of colposcopists, achieving an AUC of 0.678 for LSIL and 0.777 for HSIL. Independent validation was performed.	101,267 samples	Colposcopic Artificial Intelligence Auxiliary Diagnostic System (CAIADS):	To evaluate AI system that diagnoses colposcopy images	Xue et al., 2020 [93].
Tumor staging based on clinical and laboratory data				
Neural network, RF, and NB demonstrated superior classification ability with combined input. Accuracies of neural network, RF, and NB were 0.767, 0.718, and 0.688, respectively, and the AUCs were 0.793, 0.779, and 0.771. Neither external nor independent validation was performed.	324 patients	Decision tree, LR, SVM, RF, naïve Bayes (NB), and neural network	To diagnose lung cancer staging based on tongue images and tumor markers	Shi et al., 2023 [94].
Survival prediction based on clinical and laboratory data				
All six models demonstrated satisfactory predictive performance, with AUCs ranging from 0.73 to 0.86. The 3-year model exhibited the highest performance, achieving an AUC of 0.86. Independent validation was performed.	438 patients	Gradient boosting machine (GBM)	To estimate survival in patients with metastatic prostate cancer	Anderson et al., 2022 [95].
SVM-ELAS performed better than LR-ELAS and CART-ELAS. SVM-ELAS exhibited superior performance with an average AUC of 0.736, demonstrating significant enhancements over SVM-AdaBoost, SVM-Bagging, SVM-SMOTE, and SVM-TomekLinks. Multiple independent validations were performed.	1848 patients	SVM-ELAS, LR-ELAS, CART-ELAS	To predict survival and recurrence in patients with non-small cell lung cancer (NSCLC)	Hu et al., 2022 [96].
The GBM model demonstrated a predictive accuracy for survival with a C-index of 0.751. Independent validation was performed.	1050 patients	GBM	To predict survival in patients with intrahepatic cholangiocarcinoma after liver resection	Ji et al., 2022 [97].
The cause-specific Cox model and PLANN demonstrated the highest performance, closely followed by the Fine–Gray model, RF, and PLANN original. Independent validation was performed.	Sample size was not specified	RF; partial logistic artificial neural network (PLANN)	To predict survival with data on competing risk	Kantidakis et al., 2023 [98].
The 1-, 3-, and 5-year AUCs were 0.794, 0.849, and 0.872. Neither external nor independent validation was performed.	483 patients	RF	To predict survival of patients with urothelial carcinoma	Liu et al., 2023 [99].
A nomogram predicting 1-, 3-, and 5-year survival was created using selected LOFs and HOFs by DeepSurv, demonstrating favorable predictive efficacy for lung cancer patients at 1 and 3 years, with a C-index of 0.744. Neither external nor independent validation was performed.	1558 samples	DeepSurv: based on a neural network	To predict survival with different features from routine blood tests	Luo et al., 2023 [100].
XGBoost yielded the best outcome with the highest AUCs. XGBoost achieved an accuracy of 83% in predicting the mortality rate for Group 1 post-surgical resection and 69% accuracy for Group 2 post-trans arterial chemoembolization (TACE). Neither external nor independent validation was performed.	10,742 patients	Voting ensembles, LR, KNN, decision tree, SVM, RF, XGBoost, light GBM, and natural gradient boosting (NG Boost)	To predict mortality rates with clinical features	Noh et al., 2022 [101].
DeepSurv yielded a C-index of 0.824 using the training cohort, while validation using the test cohort yielded a C-index of 0.821. Neither external nor independent validation was performed.	49,275 patients	DeepSurv	To predict survival with SEER database	Yu et al., 2022 [102].
Recurrence prediction based on clinical and laboratory data				
For 1-year post-NAC, RF outperformed LR with AUC of 0.810. For 5-year post-NAC, RF again outperformed LR with AUC of 0.829. And for external validation set with SEER database, RF outperformed LR with AUC of 0.779. Both external and independent validation were performed.	315 patients	RF and LR	To predict breast cancer relapse or metastasis with clinical data	Jin et al., 2023 [103].
AdaBoost showed a prediction performance of a sensitivity of 0.673, specificity of 0.807, accuracy of 0.799, and AUC of 0.740. Independent validation was performed with 30% of sample.	9598 patients	SVM, LR, KNN, NB, RF, gradient boost, AdaBoost, and XGBoost	To predict recurrence in renal cell carcinoma with clinical data	Kim et al., 2022 [104].
Cancer treatment response prediction based on clinical and laboratory data				
The average accuracy from D1 to D3 in predicting outcomes on the test set was 83.21%, with specific accuracies of 83.96% for survival. The optimal DQL model (survival + dysphagia, two neural network layers, without radiomics input) demonstrated a 70.4% similarity to physician decisions on the training set and 69.65% on the test set. Neither external nor independent validation was performed.	536 patients	Deep Q Learning (DQL): based on neural network	To select treatments and their outcomes with clinical data using DQL	Tardini et al., 2022 [105].

## Data Availability

The original contributions presented in the study are included in the article; further inquiries can be directed to the corresponding author/s.
